# Quantitative assessment of intervortex anastomosis in central serous chorioretinopathy and fellow eyes: Does the size of anastomotic vessels matter for the diagnosis?

**DOI:** 10.1007/s00417-024-06517-7

**Published:** 2024-05-24

**Authors:** Sibel Demirel, Rabia Eroğlu Ayaz, Özge Yanık, Figen Batıoğlu, Emin Özmert, Claudio Iovino, Jay Chhablani

**Affiliations:** 1 Department of Ophthalmology, Ankara University School of Medicine, Vehbi Koç Eye Hospital, Mamak Street, Dikimevi, Ankara, Turkey; 2 Department of Ophthalmology, Marmara University Pendik Training and Research Hospital, Ankara, Turkey; 3 Eye Clinic, Multidisciplinary Department of Medical Surgical and Dental Sciences, University of Campania Luigi Vanvitelli, Naples, Italy; 4 Department of Ophthalmology, University of Pittsburgh, Pittsburgh, PA USA

**Keywords:** Central serous chorioretinopathy, Intervortex venous anastomosis, Choroidal vascularity index

## Abstract

**Purpose:**

To evaluate the frequency and size of intervortex anastomosis at the posterior pole on en-face spectral domain optical coherence tomography (SD-OCT) images in central serous chorioretinopathy (CSC) cases and their fellow eyes and its associations with choroidal morphology.

**Methods:**

Sixty-five treatment-naive eyes of 65 patients with CSC, 65 fellow eyes, and 55 eyes of healthy age-matched participants were included. The presence of intervortex anastomosis at the watershed zone and asymmetry of the choroidal vessels between the superior and inferior macula were evaluated using 6 × 6 mm en-face SD-OCT. The diameter of the widest Haller vessel and the diameter of the widest anastomotic Haller vessel passing through the watershed zone were measured on en-face SD-OCT images. The choroidal vascularity index (CVI) was assessed using ImageJ software.

**Results:**

Intervortex vein anastomosis on the horizontal watershed zone was detected in 75.4% diseased eyes, 61.5% in fellow eyes, and 36.4% in healthy age-matched controls (p < 0.001). The mean CVI was significantly higher in both diseased (74.3 ± 2.3%) and fellow (73.8 ± 2.2%) eyes of CSC cases than in healthy controls (72.5 ± 2.3%) (p = 0.002, p = 0.013, respectively). In the cases with intervortex vein anastomosis, the diameter of the widest anastomotic Haller vessel passing through the watershed zone was 0.40 ± 0.10 mm in diseased eyes, 0.35 ± 0.11 mm in fellow eyes, and 0.30 ± 0.09 mm in healthy age-matched controls (p = 0.001).

**Conclusions:**

Intervortex anastomosis might be seen as a variation in normal eyes, however, its frequency and the size of anastomotic vessels are significant higher in not only CSC but also in fellow eyes.

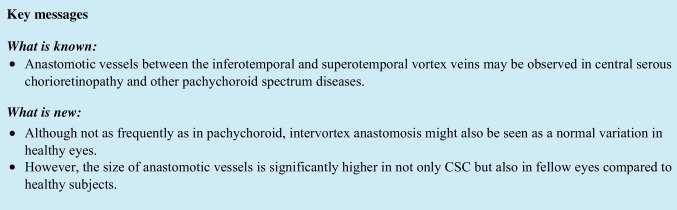

## Introduction

The term "pachychoroid" is defined as choroidal thickening with dilatation of the external choroidal vessels [1]. The vascular anatomy and physiology of the choroidal vascular system are altered in pachychoroidal conditions [2]. Numerous theories have been suggested, but the pathophysiology of disorders in the pachychoroid spectrum is still unknown. Central serous chorioretinopathy (CSC) is one of these pachychoroid spectrum diseases. CSC is a common chorioretinopathy that causes serous detachment of the retina secondary to leaks from the level of the retinal pigment epithelium (RPE) caused by the choroidal hyperpermeability.

Recently, advances in optical coherence tomography (OCT) technology, including enhanced depth imaging (EDI) mode and swept source devices, have enabled a more detailed visualization of the choroid. It was shown that the eyes of the CSC patients had thick choroid, and dilatation of the large choroidal vessels was detected as a cause of the increased thickness [3]. Indocyanine green angiography (ICGA)shows delayed filling of the choriocapillaris in the early phase and hyperpermeability of the choroidal vessels in the mid-late phases supporting the hypothesis that hydrostatic pressure in the choroid might be a cause of CSC [4, 5]. Recently, it was suggested that venous outflow problems might be the reason. Normally, the posterior ciliary artery branches into the medial and lateral posterior ciliary arteries and extends to the choriocapillaris [6]. Regions between adjacent branches are defined as physiological and, collected by the venules and drained into the larger vessels. These vessels continue directly into the ampulla of the vortex vein without anastomosis with each other. Vortex veins generally range from 3 to 8, and 65% of people have 4 or 5 veins [7]. They appear to be symmetrical quadrants, although there are multiple vortex veins at the beginning of each quadrant [8]. Studies on the vascular physiology of the choroid suggest that there may be abnormalities in the local control of this venous outflow. These vascular changes include abnormal amounts of remodeling and anastomosis formation. Using en-face OCT images of the choroid, an asymmetrical pattern of superior and inferior vortex vein vessels has been reported in the CSC [9]. In a study published in 2020, Spaide et al. introduced the venous overload choroidopathy hypothesis to describe various choroid diseases of the eye and to support the existing literature. Anastomotic vessels between the inferotemporal and superotemporal vortex vein systems were observed in central serous chorioretinopathy and other pachychoroid spectrum diseases [6]. Several clinical studies have revealed that vortex vein anastomosis may be a compensatory mechanism for vortex choroidal congestion. Matsumoto et al. highlighted that anastomosis can be observed in the healthy control group, but it is significantly more common in pachychoroid spectrum diseases compared to healthy controls [10]. Similarly, Bacci et al. reported that intervortex venous anastomosis was 88.5% in patients with pachychoroid disease and 34.6% in control eyes [11].

The observation of pachyvessels, increase in the size of the lumen of outer choroidal vessels associated with venous overload choroidopathy and impingement on the choriocapillaris layer subsequently leads to low-flow signals or flow-voids observed on OCTA [12, 13]. As a consequence of ischemia of the choriocapillaris, there is increase in angiogenic cytokines that result in macular neovascularization [8]. With the advent of OCTA, there is increased number of neovascular CSCR patients (about 1 out of 5 CSCR patients) [14]. Recent studies reported that OCTA has higher sensitivity and specificity than dye angiography in diagnosing neovascular CSCR [14, 15]. Clinically when performing OCTA for the investigation of CSCR eyes, it is imperative to examine for the choriocapillaris flow-voids and one should not overlook any secondary macular neovascularization because it is crucial towards making therapeutic decisions for the best possible clinical outcome.

As we all know, even the fellow eyes of the CSC cases have thicker choroid [16], dilated choroidal vessels and hyperreflective areas identified on swept source OCTA [17]. Therefore, determining whether there is intervortex anastomosis in the fellow eyes will strengthen the presence of intervortex anastomosis as a common feature of the pachychoroid spectrum. New imaging features are needed as this feature can also be found in healthy eyes.

This study aimed to evaluate the frequency and differences in the size of intervortex anastomotic vessels at the posterior pole in CSC and fellow eyes on en-face spectral domain OCT (SD-OCT) and their associations with choroidal morphology, extend of the RPE alteration area on fundus autofluorescence (FAF), and leakage pattern on fluorescein angiography (FA). To the best of our knowledge, this is the first study to quantitatively evaluate intervortex anastomosis and anastomotic vessel diameters in both fellow eyes of the CSC eyes and healthy controls.

## Materials and methods

### Patient selection

This retrospective comparative study included 65 eyes of 65 patients with treatment-naive active acute or chonic CSC, diagnosed at Ankara University Faculty of Medicine between April 2017 and February 2022. Fifty-five healthy age-matched individuals were included in the study as the control group. Eyes with CSC (group 1) and their fellow eyes (group 2) were included in this analysis. The control group (group 3) consisted of healthy individuals without any history of ocular or systemic disease.

The treatment-naive patients diagnosed with active CSC were included in the study. Presence of serous detachment including the fovea on spectral domain OCT (SD-OCT), hyperfluorescent changes typical of CSC on ICGA including dilated choroidal vessels, choroidal filling defects, choroidal venous dilation and vascular congestion, and choroidal vascular hyperpermeability on mid to late phase, and ill‐defined hyperfluorescent leaks (focal or multifocal leakage) at the level of RPE on FA or diffuse oozing of dye due to diffuse RPE defect resulting in patches of granular or mottled hyperfluorescence in mid and late phase FA were defined as inclusion criteria [1, 18].

The exclusion criteria were history of intraocular inflammation and ocular trauma, any additional chorioretinal disease, glaucoma, media opacities, or artefacts preventing adequate imaging, high refractive error (spheric equivalent ≥ 5D), previous intravitreal anti-vascular endothelial growth factor therapy, previous photodynamic therapy, and previous vitreoretinal surgery. Additionally, cases with pigment epithelial detachments were excluded from the study as they could lead to shadowing artifacts.

### Clinical evaluation and imaging

The participants’ medical charts were reviewed to collect demographic and clinical data. All participants underwent a detailed ophthalmologic examination including best corrected visual acuity (BCVA) assessment using ETDRS charts, slit-lamp biomicroscopy, applanation tonometry, and a dilated fundus examination. Multimodal fundus imaging including Spectral domain OCT (SD-OCT) (Spectralis HRA + OCT; Heidelberg Engineering, Heidelberg, Germany), OCTA (AngioVue software, RTVue XR Avanti, Optovue, Inc., Fremont, CA), FAF, FA, and ICGA (HRA 2 Heidelberg Engineering) were performed in each CSC case. For the healthy control group, SD-OCT and OCTA images were performed. Due to the routine practice in our clinic, all measurements were performed between 10:00 and 12:00 in the morning following pupil dilation and after a rest period of at least half an hour.

Patients with CSC were divided into two groups according to FA leakage characteristics (focal vs diffuse leakage). The area of involvement was classified primarily based on FAF imaging. RPE alterations ≤ 2-disk diameters were defined as simple CSC, while > 2-disk diameter was classified as complex CSC [19]. Choroidal hyperpermeability was noted at the 5–10 min on ICGA in all CSC and fellow eyes.

Both the presence of intervortex anastomosis passing through at the watershed zone and also asymmetry of the choroidal vessels between the superior and inferior macula were evaluated using 6 × 6 mm en-face SD-OCT (RTVue XR Avanti, Optovue) [10]. En-face OCT scans were obtained using HD Angio Retina 6 × 6 mm mode of AngioVue OCTA software of RTVue XR Avanti (Optovue, Inc., Fremont, CA). This 70,000 Hz (840-nm wavelength) system provides a high scanning density (400 × 400). Due to the individual variability of the choroidal thickness and Haller’s layer position in each subject, segmentation was individualized. The technique described by Savastano et al. was used for individualized segmentation with minor modifications [20]. A 40-micron parallel plane achieving best detectable and widest vessels in Haller's layer below the RPE was selected to generate an appropriate Haller’s layer imaging. The diameter of the widest Haller vessel on en- face SD-OCT was measured by drawing a 90-degree perpendicular line to the wall of the largest Haller vessel on the 6 × 6 image using ImageJ software. Horizontal B-scan EDI-OCT images through the fovea were used to measure subfoveal choroidal thickness (SFCT). ImageJ program version 1.52u bundled with 64-bit Java 1.80_112 (Wayne Rasband, National Institutes of Health, Bethesda, Maryland, USA, https://imagej.nih.gov/ij) was used for the measurement of CVI on the horizontal SD-OCT B scan passing through the fovea. The vertical margins of the choroid were selected from the RPE to the choroidoscleral border. Niblack auto local thresholding was applied for binarization. The dark regions were defined as luminal choroidal area and the bright regions as stromal choroidal area. The CVI, the percentage of the luminal choroidal area to the total choroidal area, was calculated [21].

The primary outcome measure was the determination of the presence of intervortex anastomosis among all groups. The secondary outcome measure was alterations in the CVI, highest vessel diameter passing the watershed zone, and measurement of the largest vessel diameter in the image.

### Statistics

Statistical analyses were performed using the Statistical Package for Social Sciences, version 15.0. In the study, descriptive statistics were presented as mean ± standard deviation for quantitative variables with a normal distribution, median (min–max) for variables with non-normal distribution, and percentages for qualitative variables. When the number of independent groups was 2, the relationship between the groups in terms of a numerical variable was examined using the "Significance of the Difference Between Two Means Test" when the parametric test assumptions were met, and the "Mann–Whitney U Test" if the parametric test assumptions were not met. Chi-Square Test or Fisher's Exact Test was used in the analysis of qualitative data. The relationship between more than 2 groups in terms of a variable specified by the measurement was evaluated by One-Way Analysis of Variance or its non-parametric counterpart, Kruskal–Wallis Analysis of Variance. A value of p < 0.05 was considered statistically significant.

## Results

The clinical characteristics of patients with CSC and healthy controls are presented in Table 1.The patients with CSC included 47 males (72.3%) and 18 females (27.7%). Their mean age was 45.3 ± 7.4 (30–60) years. The healthy control group included 39 males (70.9%) and 16 females (29.1%). Their mean age was 46.5 ± 9.8 years (p = 0.914). The mean manifest refractive spherical equivalents were—0.35 ± 0.61 D in fellow eyes,—0.12 ± 0.63 in CSC eyes, and -0.41 ± 0.57 D in healthy controls (p = 0.213).

**Table 1:** Comparisons of the mean subfoveal choroidal thickness, choroidal vascularity index, diameter of the widest Haller vessel, diameter of the anastomotic Haller vessel passing in watershed zone, and the frequency of intervortex anastomosis between the groups

Participant Characteristic n = 120				P values			
				Pairwise Comparisons*			
Study Variables	Group 1 (CSC eyes) n = 65 Mean ± SD	Group 2 (Fellow eyes) n = 65 Mean ± SD	Group 3 (Control) n = 55 Mean ± SD	Kruskal–Wallis Test	Group 1vs Group 2	Group 2 vs Group3	Group 1 vs. Group 3
Subfoveal choroidal thickness (μm)	416.9 ± 94.8	375.6 ± 90.3	286.0 ± 66.8	** < 0.001**	= 0.073	** < 0.001**	** < 0.001**
CVI (%)	74.3 ± 2.3%	73.8 ± 2.2%	72.5 ± 2.3%	** = 0.001**	** = **1.000	** = 0.013**	**0.002**
The diameter of the widest Haller vessel (mm)	0.45 ± 0.09	0.42 ± 0.09	0.33 ± 0.06	** < 0.001**	** < 0.001**	= 0.210	** < 0.001**
The diameter of the anastomotic Haller vessel passing in watershed zone mm	0.40 ± 0.10	0.35 ± 0.11	0.30 ± 0.09	**0.001**	= 0.137	= 0.159	**0.001**
Intervortex anastomosis ( +)	75.4%	61.5%	36.4%	** = 0.001**	= 1.000	** = 0.013**	**0.002**

^*^Dunn’s test results for Kruskal–Wallis Test

Bold values indicate statistically significant p values. CSC: Central Serous Chorioretinopathy, CVI: Choroidal vascularity index

**Fig. 1:**
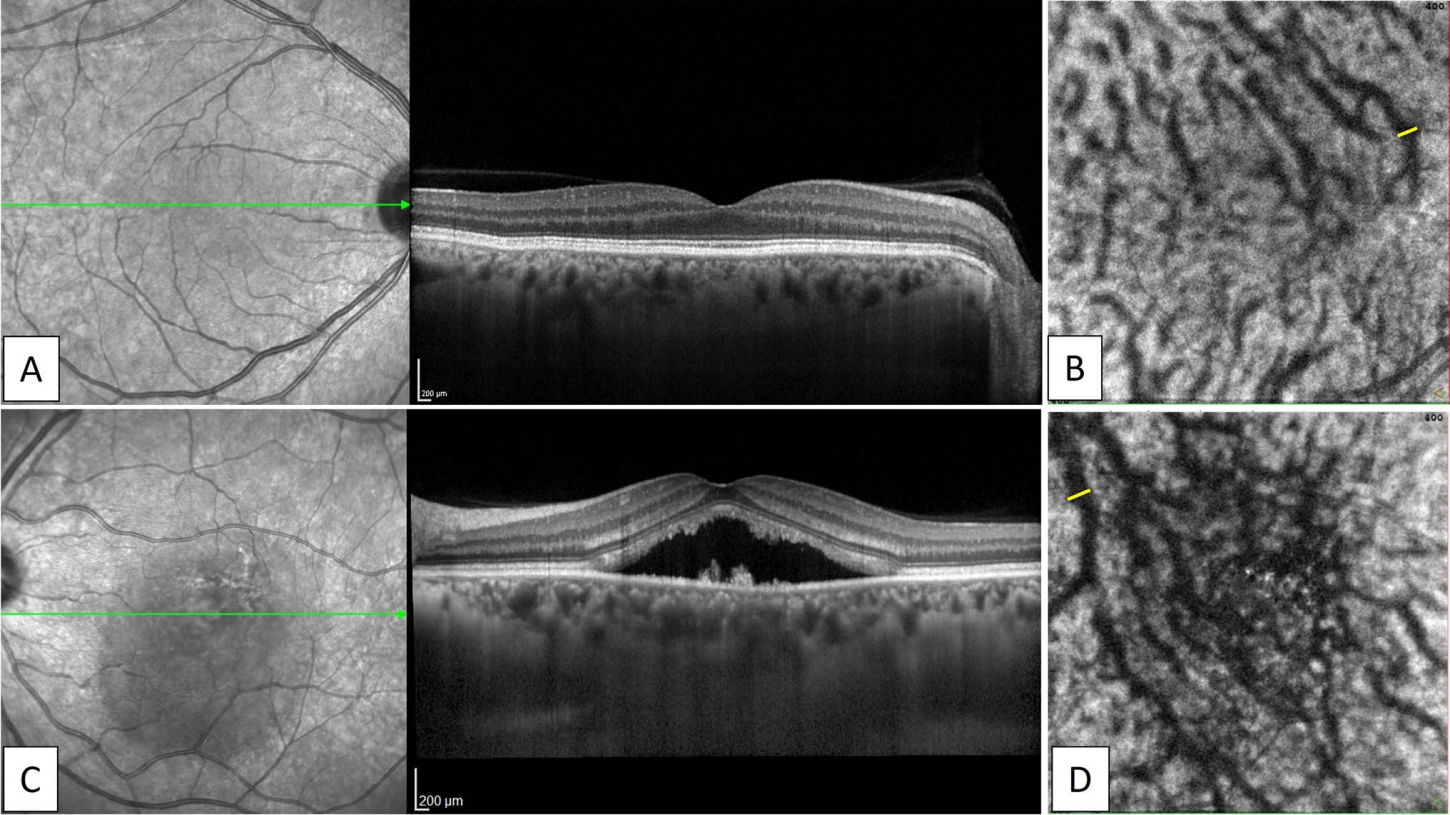
Images of a 49-year-old man with central serous chorioretinopathy in his left eye. A) A horizontal EDI-OCT image through the fovea in his right eye revealed normal appearance of the retina with dilated outer choroidal vessels. B) En-face OCT image (6 × 6 mm) through the fovea showed superior and inferior vortex veins are symmetrical, and the horizontal watershed zone through the macula is preserved. C) A horizontal EDI-OCT image through the fovea in his left eye revealed subretinal fluid and pachychoroid with dilated outer choroidal vessels. D) En-face OCT image (6 × 6 mm) through the fovea showed dilated vortex veins in the deep layer of the choroid. Horizontal watershed is lost because of the anastomoses between the superior and inferior vortex veins. The diameters of the widest Haller vessels are marked with a yellow line on the en-face OCT images (B, D)

**Fig. 2:**
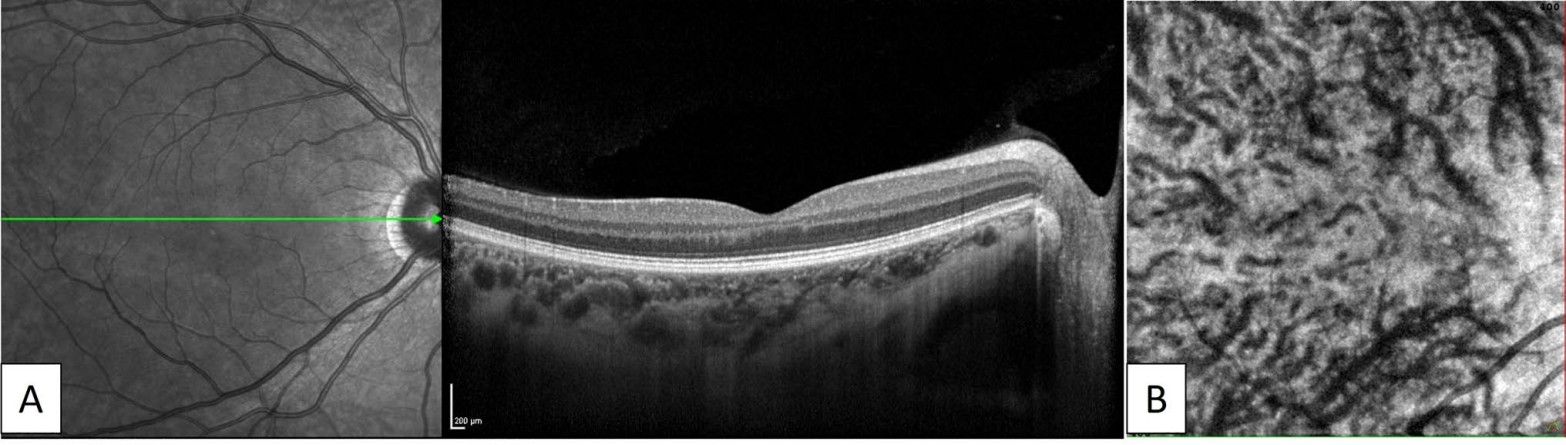
Images of the healthy left eye of a 46-year-old man in the control group. A) A horizontal EDI-OCT image through the fovea revealed normal appearance of the retina and choroid. B) En-face OCT image (6 × 6 mm) through the fovea showed superior and inferior vortex veins are symmetrical, and the horizontal watershed zone through the macula is preserved

**Fig. 3:**
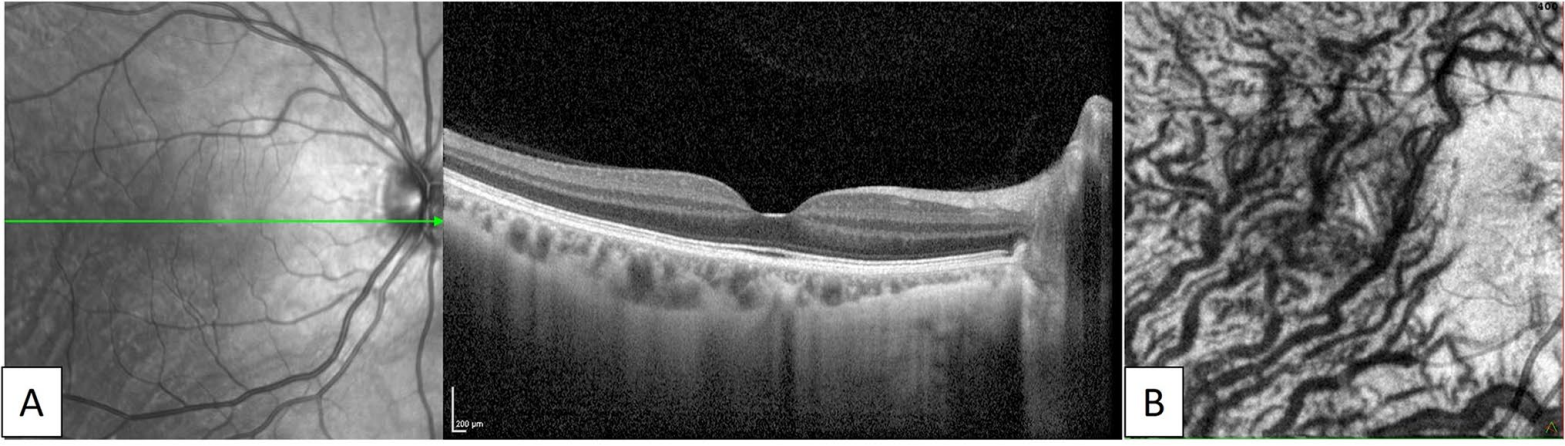
Images of the healthy right eye of a 47-year-old woman in the control group. A) A horizontal EDI-OCT image through the fovea revealed normal appearance of the retina and choroid. B) En-face OCT image (6 × 6 mm) through the fovea showed dilated vortex veins in the deep layer of the choroid. Horizontal watershed is lost because of the anastomoses between the superior and inferior vortex veins

Choroidal hyperpermeability on ICGA was observed in all diseased eyes and 73.8% of the fellow eyes. Intervortex vein anastomosis was detected in 75.4% diseased eyes (Fig. 1), 61.5% in fellow eyes, and 36.4% in healthy age-matched controls (Figs. 2 and 3) (p < 0.001). The mean SFCT was 416.9 ± 94.8 μm in diseased eyes, 375.6 ± 90.3 μm in fellow eyes, and 286.0 ± 66.8 μm in healthy age-matched controls (p < 0.001). The mean CVI value was significantly higher in both diseased (74.3 ± 2.3%) and fellow (73.8 ± 2.2%) eyes of CSC cases than in healthy controls (72.5 ± 2.3%) (p = 0.002, p = 0.013, respectively). The diameter of the widest Haller vessel on en-face OCT was 0.45 ± 0.09 mm in diseased eyes, 0.42 ± 0.09 mm in the fellow eyes, and 0.33 ± 0.06 mm in the healthy age-matched controls (p < 0.001). In the cases with intervortex vein anastomosis, the diameter of the widest anastomotic Haller vessel passing through the watershed zone 0.40 ± 0.10 mm in diseased eyes, 0.35 ± 0.11 mm in fellow eyes, and 0.30 ± 0.09 mm in healthy age-matched controls (p = 0.001). RPE alterations ≤ 2-disk diameters were 80% and > 2-disk diameters were 20% in diseased eyes. Patients were divided into two groups according to FA (focal vs. diffuse leakage). Focal leakage was 18.5% and diffuse leakage were 81.5% in the diseased eyes.

Comparisons of the extend of the RPE alteration area, FA leakage pattern, mean CVI, mean SFCT, and mean the diameter of the widest Haller vessel between the patients with intervortex anastomosis and without intervortex anastomosis is given in Table 2. RPE alterations ≤ 2-disk diameters were 79.6% in the patients with intervortex vein anastomosis and 81.2% in the patients without anastomosis (p = 1.00). Regarding FA leakage patterns, focal leakage was observed in 16.3% and diffuse leakage were observed in 83.7% of the cases with intervortex vein anastomosis, whereas focal leakage was detected in 25% and diffuse leakage were detected in 75% of the cases without intervortex vein anastomosis (p = 0.47). The mean SFCT was 418.6 ± 88.0 μm in the patient with intervortex anastomosis and 411.8 ± 116.3 μm in the without intervortex anastomosis group (p = 0.95). The mean CVI value was (74.3 ± 2.5%) in the patient with intervortex anastomosis and (74.2 ± 1.6%) in the without anastomosis patient group (p = 0.90). The diameter of the widest Haller vessel 0.46 ± 0.09 mm in the patient with intervortex anastomosis and 0.43 ± 0.07 mm in the without anastomosis patient group (p = 0.17).

**Table 2:** Comparisons of the retina pigment epithelium alterations, fluorescein angiography leakage pattern, choroidal vascularity index, subfoveal choroidal thickness, the diameter of the widest Haller vessel between the patients with intervortex anastomosis and without intervortex anastomosis

	Group 1 with anastomosis n = 49 n (%) / Mean ± SD	Group 2 without anastomosis n = 16 n (%) / Mean ± SD	P value
RPE alterations			
• RPE alterations ≤ 2-disc diameters	39 (79.6%)	13 (81.2%)	1.00
• RPE alterations > 2-disc diameters	10 (20.4%)	3 (18.8%)	
FA leakage pattern			
• Focal	8 (16.3%)	4 (25.0%)	0.47
• Diffuse	41 (83.7%)	12 (75.0%)	
CVI (%)	74.3 ± 2.5	74.2 ± 1.6	0.90
SFCT (μm)	418.6 ± 88.0	411.8 ± 116.3	0.95
The diameter of the widest Haller vessel (mm)	0.46 ± 0.09	0.43 ± 0.07	0.17

CVI: Choroidal vascularity index. FA: Fluorescein angiography. RPE: Retina pigment epithelium. SFCT: Subfoveal choroidal thickness

In the diseased eyes, a statistically significant very strong positive correlation was detected between the diameter of the anastomotic Haller vessel passing through the watershed zone and the diameter of the widest Haller vessel (r = 0.791, p < 0.001). In addition, CVI values were significantly correlated with the diameters of the anastomotic Haller vessel passing through the watershed zone (r = 0.322, p = 0.024). In fellow eyes, the diameter of the anastomotic Haller vessel passing through the watershed zone was significantly correlated with CVI (r = 0.386, p = 0.014), subfoveal choroidal thickness (r = 0.332, p = 0.036), and the diameter of the widest Haller vessel (r = 0.686, p < 0.001). In control eyes, a statistically significant very strong positive correlation was detected between the diameter of the anastomotic Haller vessel passing through the watershed zone and the diameter of the widest Haller vessel (r = 0.703, p = 0.001).

## Discussion

In this study, we found that choroidal hyperpermeability on ICGA, increased thickness of the choroid, and CVI values were observed in diseased eyes and most of the fellow eyes in patients with CSC. The intervortex vein anastomosis was a common finding not only in diseased eyes but also in fellow eyes, and although less common, it can be seen as a normal variation in healthy eyes. However, on en-face OCT, the diameter of the widest Haller vessel in the macular area and the diameter of the widest anastomotic Haller vessel passing through the watershed zone were greater in CSC eyes compared to the healthy control group. As expected, the diameter of the Haller vessel that anastomoses with the watershed zone was strongly linked to CVI. The same was true for the diameter of the widest Haller vessel in the macula of both CSC eyes and their fellow eyes.

We looked into the anastomosis and even more details of the anastomotic vessels to see if there were any similarities between CSC and their fellow eyes or any differences between diseased eyes and the healthy control group. This could help us figure out how venous outflow overload, which is the proposed mechanism recently, can affect the structure of the outer chorodal vessels in pachychoroid eyes. And could anastomosis itself be a good marker for the differential diagnosis of pachychoroid disease? You can see anastomosis in healthy eyes as well as in eyes with age-related macular degeneration. This doesn't mean that anastomosis is always a sign of venous overload or that venous overload has to end with anastomosis [22]. In order to clear this up, the anastomotic vessels could be looked at in terms of their size, whether they taper or don't taper in the watershed zone, and the difference in the size of the choroidal vessels between the upper and lower macular areas. Hiroe et al. found that an asymmetric vortex vein was present in 38% of normal subjects [22]. This asymmetry was present in all eyes of the patients with CSC. Dominant vortex veins were dilated markedly in CSC. Congestion of the dominant vortex veins might enhance the permeability of fenestrated choriocapillaris in the macular region. Asymmetric dominant vortex veins appear to be a predisposing factor for CSC. It has been reported that, as a disease, CSC was not a unilateral pathology in most of the cases [23]. Even manifest subretinal fluid might not be seen in the fellow eyes; retina pigment epithelial abnormalities or choroidal hyperpermability have been found in over 90% of the fellow eyes [23]. Additionally, it was reported that the hyporeflective ratio (luminal ratio) increased in the outer choroidal layer in the fellow eyes of CSC patients [16]. However, macular vortex anastomosis and its pattern in the fellow eyes of CSC patients were never mentioned. Accordingly, we found that the choroidal hyperpermability on ICGA, the thickness of the choroid, the CVI values, and the ratio of anastomosis seen in the macular area are all pretty much the same between CSC and their fellow eyes. The size of the largest vessels in the macular area and the size of the largest anastomotic vessels are strikingly similar in size between CSC and fellow eyes, although they are statistically larger than the mean size of these vessels in the healthy age-matched control group. However, the diameter of the widest Haller vessel and the anastomotic Haller vessels did not show a statistically significant difference between control eyes and fellow eyes, even if the number of anastomoses is higher in the fellow eyes of CSC. The only statistically significant difference among CVI, CCT, intervortex anastomosis, the diameter of the widest anastomotoic Haller vessel, and the diameter of the widest Haller vessel in the macular area between CSC and fellow eyes was the diameter of the widest Haller vessel in the macular area. It makes sense that the higher the degree of venous outflow problems, the more dilated Haller vessels are in the watershed zone or macular area, which can explain the imaging features of the CSC eyes. We believe that it strongly advocates venous outflow resistance in pachychoroid disease. It is likely that the degree of the venous outflow problem, its effect on intervortex venous anastomosis, and finally the amount of pressure in the choroidal vessels located in the macular area, which can be depicted not only as anastomosis but also the diameter of vessels, may have a role in determining the clinical spectrum of this disease, such as pachychoroid pigment epiteliopathy, or subretinal fluid and CSC.

The assessment of the vortex veins may also be relevant to understanding the pathophysiology of the pachychoroid disease spectrum, particularly since there is increasing evidence suggesting choroidal venous congestion as a common pathogenic background to this complex clinical phenotype [10]. Bacci and colleagues reported that eyes with pachychoroid disease showed a significant within-subject variance in the proportion of the postequatorial fundus drained by each vortex vein system, which is not a feature in healthy control eyes in a study using wide-angle ICGA [11]. It might be a biomarker for the diagnosis of the disease; however, both using indocyanine green and having an ultra-wide field viewing system might not be practical in clinical practice. Researchers have also utilised artificial intelligence to evaluate ultra-widefield ICGA images for classifying pachychoroid disease and to differentiate them from other pathologies, such as age-related macular degeneration, by using the associated engorgement of the outflow channels [24]. Last but not least, there are a lot more intervortex venous anastomoses in this area of the eye between the dilated superior and inferior vortex veins. So, Spaide and his colleagues found that the central macula is where most anastomoses happen in eyes with CSC and pachychoroid-associated neovascularization [8]. So, studying the macular area and using binarization techniques on non-invasive imaging methods like en-face OCT might make it easier to understand the choroidal vasculature in the macular area. More research is needed to show if this method is better than wide-angle ICGA. One of the drawbacks of this retrospective study is the lack of wide-field ICGA and en-face swept source OCT to better evaluate the anastomosis between the quadrans. However, in clinical practice, understanding whether there is an anastomosis in the watershed zone or not is tricky with dye angiography because of the leaky choroidal vessels and stromal staining. As a result, we believe that evaluating the macula with en-face OCT slabs may provide some clues about etiopathogenesis. Similar to our result, Cheung and colleagues evaluated vascular structures in the choroidal layers of the patients with neovascular age-related macular degeneration (nAMD) and polypoidal choroidal vasculopathy (PCV), and they concluded that choroidal vascular remodeling is common in both AMD and PCV but may be driven by different stimuli [25]. They reported a retrospective study evaluating baseline ICGA regarding choroidal vessel morphology such as macular anastomosis, dilated Haller veins, and focal variation in vessel caliber in eyes with typical nAMD and PCV. It was found that even if macular anastomosis was common in both diseases, dilated Haller veins were numerically less common in typical nAMD than PCV, and vascular caliber variation was numerically more common in typical nAMD than PCV. The presence of all three features was more common in eyes with PCV compared with typical nAMD. In a multivariable analysis, every increase of 100 mm of choroidal thickness conferred a 2.75 risk of having all three features present [25]. Similar to their results, the strong correlation between the diameter of the anastomotic Haller vessel passing through the watershed zone and the diameter of the widest Haller vessel in the macular area makes us think that the diameter of the vessel might be an indirect indicator of venous outflow problems not only in diseased eyes but also in fellow eyes of pachychoroid disease, and it may help us understand different situmuli for the anastomosis of the macula. We do not know the exact driving factor for macular anastomosis in different diseases or the reason for the anastomosis in healthy eyes. They claimed that the stimulus of the need for anastomosis may be different for AMD, such as atherosclerosis and venous overload for the pachychoroid disease. We can conclude that, even though we didn't study PCV eyes in the same way as Cheung and colleagues, since PCV and pachychoroid eyes may represent the same spectrum of the disease, factors such as the size of the anastomotic vessels and focal choroidal vessel dilating in favor of AMD may provide a clue for the correct diagnosis in addition to the presence of the anastomosis between vortex veins.

The major limitations of the study were its retrospective nature and cross-sectional single-center design. Additionally, the use of 6 × 6 mm en-face OCT images for the visualisation of the intervortex anastomosis is one of the limitations leading to the imaging of a restricted area. However, Hiroe et al. showed that asymmetric vortex veins serve the macular region or posterior pole in approximately 87.0% of the CSC cases [22]. Therefore, we believe that this situation do not have a significant impact on the study results. Despite all these mentioned limitations of the study, we observed significant morphological and numerical differences between the groups.

In conclusion, intervortex anastomosis might be seen as a variation in the healthy eye; however, its frequency and the size of anastomotic vessels are more striking findings in not only CSC but also fellow eyes.
